# Early Adjuvant Medication With the mTOR Inhibitor Sirolimus in a Preterm Neonate With Compressive Cystic Lymphatic Malformation

**DOI:** 10.3389/fped.2020.00418

**Published:** 2020-07-28

**Authors:** Marion Honnorat, Loïc Viremouneix, Sonia Ayari, Laurent Guibaud, Karen Coste, Olivier Claris, Marine Butin

**Affiliations:** ^1^Service de Réanimation Néonatale, HFME, Hospices Civils de Lyon, Bron, France; ^2^Centre de Compétence des Anomalies Vasculaires Superficielles FAVA-Multi Network, HFME, Hospices Civils de Lyon, Bron, France; ^3^Service de Radiologie Pédiatrique, HFME, Hospices Civils de Lyon, Bron, France; ^4^Service de Chirurgie ORL, HFME, Hospices Civils de Lyon, Bron, France; ^5^Service de Réanimation Pédiatrique et Néonatale, CHU ESTAING, Clermont-Ferrand, France; ^6^Université Clermont Auvergne, CNRS UMR 6293, INSERM U1103, GReD, Clermont-Ferrand, France; ^7^Université Claude Bernard, Villeurbanne, France; ^8^INSERM U1111, Centre International de Recherche en Infectiologie, Equipe “Pathogénèse des Infections à Staphylocoques”, Lyon, France

**Keywords:** lymphangioma, preterm, sirolimus, sclerotherapy, mTOR inhibitor

## Abstract

Cystic lymphatic malformations result from an abnormal embryological development of the lymphatic structures. Here we report on a case of a preterm female baby, born at 34 weeks of gestation, with a voluminous cervicofacial cystic lymphatic malformation responsible for an airway obstruction. An mTOR inhibitor, sirolimus, was started from the first day of life, and was combined with iterative sclerotherapy procedures. This case illustrates a safe and successful early administration of sirolimus in a preterm neonate.

## Background

The mTOR signaling pathway is involved in cell proliferation, and is also a regulator of immune response, implicated in a vast number of diseases (1). Inhibitors of mTOR as sirolimus have long been indicated as anti-transplantation rejection medication, but recently several studies have also reported its use in the management of vascular malformations and in particular cystic lymphatic malformations (2).

Cystic lymphatic malformations result from an abnormal embryological development of the primordial lymphatic structures and are frequently related to PIK3CA mutations (1, 3) and half of them are discovered at birth (4). Approximately 75% of all cases occur in the head and neck region (4) and can thus be challenging to manage due to life-threatening complications, especially airway compression. However, there are no guidelines concerning the treatment that is therefore individualized for each patient and situation (5). In the recent literature, off-label administration of sirolimus has been reported in such indication and could constitute an effective treatment (6, 7).

There is a limited number of reports describing the use of sirolimus in newborns in particular in preterm infants. Here we report on the case of a preterm newborn with a cervicofacial cystic lymphatic malformation successfully treated with oral sirolimus.

## Case Presentation

A female preterm infant was born by cesarean section during spontaneous preterm labor at 34 weeks of gestation, from a spontaneous dizygotic twin pregnancy with a prenatal diagnosis of a cystic lymphatic malformation. Fetal MRI (Magnetic Resonance Imaging) examination showed a cystic lymphatic malformation located in the left cervical and laryngeal region and surrounding the trachea. No malformation was diagnosed in her twin sister.

The patient presented with an Apgar score of 4/5/6 and a birth weight of 2,250 g. She developed an immediate respiratory distress syndrome and ventilation was first provided by a T-piece resuscitator. Because of the airway obstruction, the ventilation was rapidly performed in lateral then in ventral decubitus position. The intubation was difficult due to infiltration and distortion of the larynx and tracheal compression and was performed at 1.5 h of life with the assistance of a video laryngoscope. Then the baby was mechanically ventilated without difficulty. The chest X-ray was normal.

**Figure 1:**
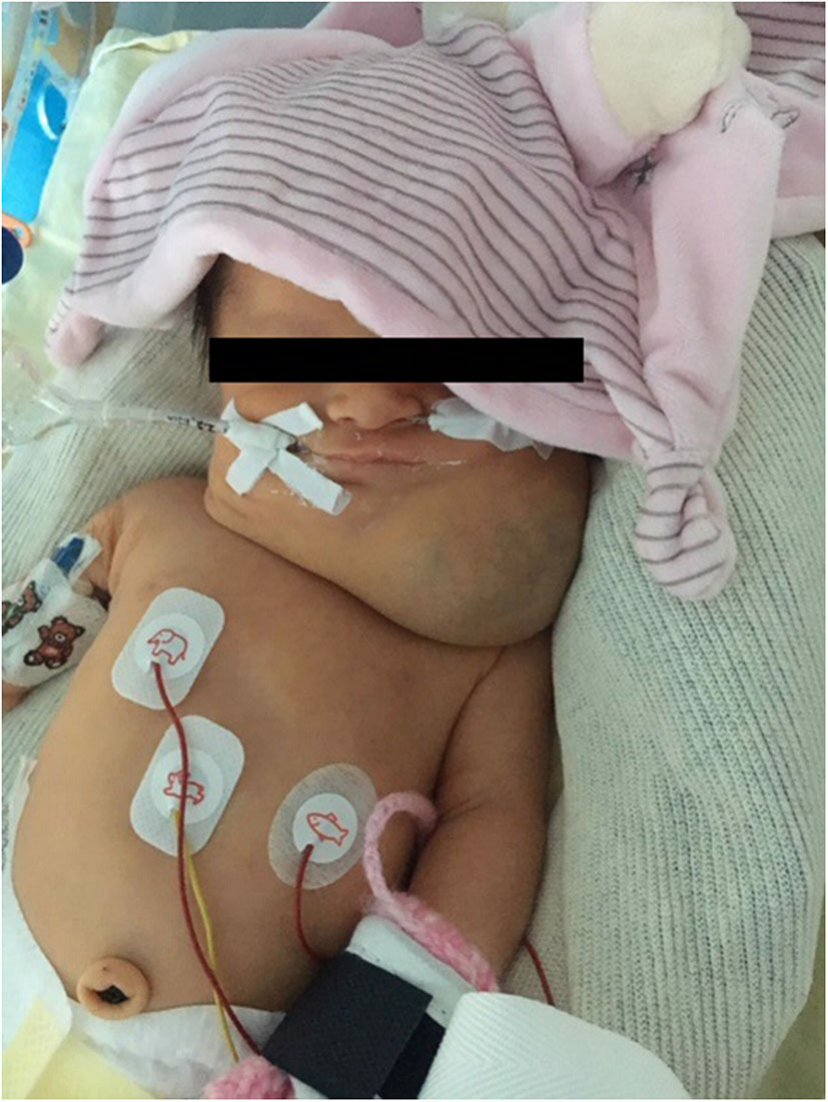
Photograph illustrating the clinical aspect of the cervical mass at 1 day of life.

**Figure 2:**
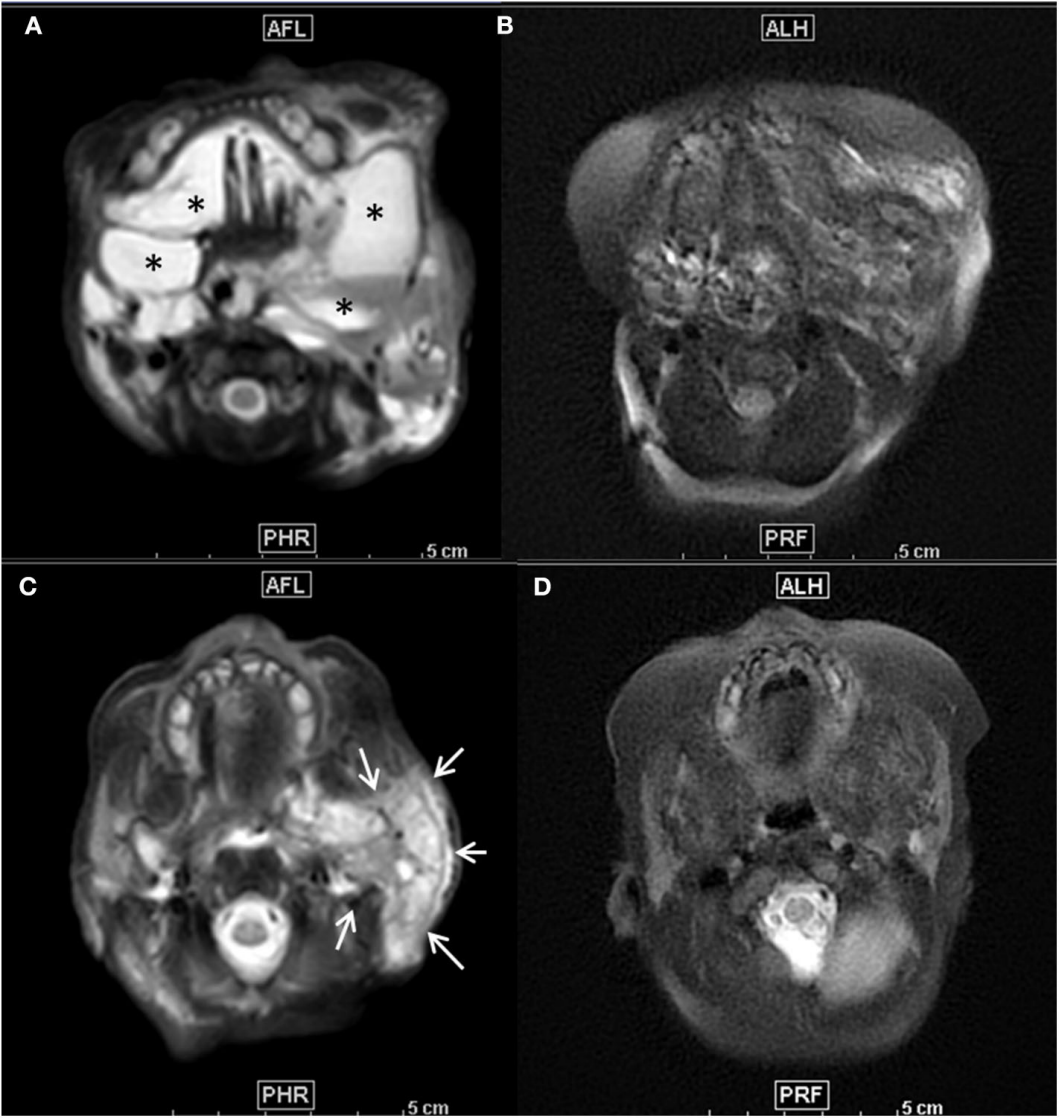
**(A–D)** Axial T2-weighted fat suppressed MR images before treatment **(A,C)** showed mixed macro- and microcystic extensive cervico-facial lymphatic malformation with diffuse involvement of the sublingual space and both cheeks by large macrocysts (*) **(A)** and extensive infiltration of the left parotid space by a large microcystic component, as demonstrated by the diffuse T2 hyperintensity of the whole left parotic space (arrows) **(C)**. Seven months after initiation of the Sirolimus and after 3 percutaneous procedures of sclerotherapy, follow-up axial T2 weighted images at the same anatomical levels **(B,D)** demonstrated a major decrease of both macrocysts **(B)** and microcystic infiltration as demonstrated by the important decrease of T2 intensity of the parotid space **(D)**.

Physical examination revealed a voluminous left cervical and facial mass (Figure 1), but no sign of dysmorphia nor abnormalities in her chest, heart, abdomen, genitalia, and back. On day 1 of life, an MRI examination showed a voluminous mass with multiple cysts invading the right parotid gland, left temporal muscle, tongue muscle, larynx and pharynx, resulting in a contralateral glottis deviation (Figures 2A,C).

Percutaneous sclerotherapy was performed at day 1 using Doxycyline 200 mg as sclerosing agent. During the procedure a large amount of blood was punctured within the cysts, preventing complete aspiration to avoid any risk of acute hypovolemia. Given the voluminous size of the mass and the need for mechanical ventilation, advice from our referring center for vascular malformations was sought. It was decided to start as of day 1 an adjuvant therapy with oral sirolimus administered twice daily at the dosage of 0.1 mg per kg per day.

**Figure 3:**
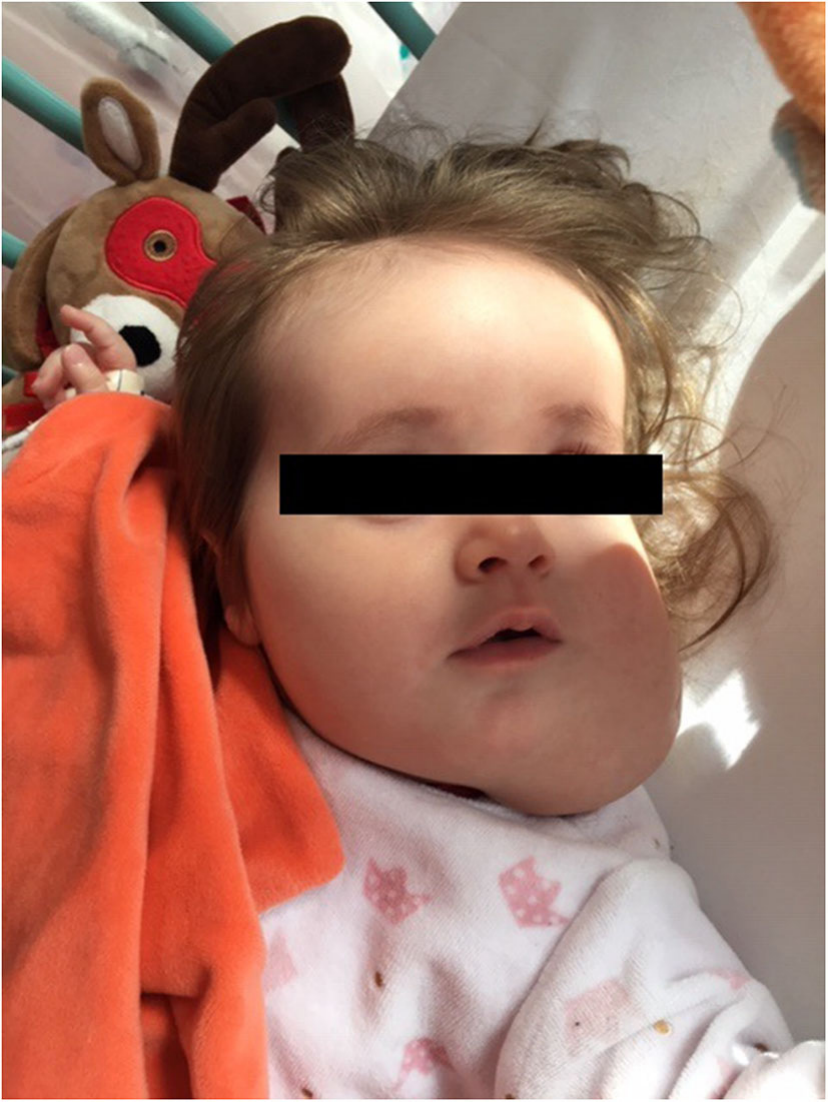
Photograph illustrating the clinical aspect of the cervical mass at 12 months of life.

After 1 month of treatment the mass size began to decrease. She underwent 3 additional percutaneous sclerotherapy procedure at 2 months old (mo), 3.5 mo, and 10 mo, using Doxycycline 186, 100 mg and finally Bleomycine 0.5 mg/kg as sclerosing agents, respectively. At 7 mo a second MRI scan was performed (Figures 2B,D) and attested the decrease of the mass. The ultrasound performed during the last procedure showed the persistence of only one macrocyst located in the left mandibular region. Clinically the mass seemed to gradually reduce in size (Figure 3). The therapy with sirolimus is still ongoing.

Respiratory evolution was marked by 2 failures of extubation. She was finally extubated at 2 mo, but she required high flow nasal cannula during the night until 9 mo due to hypercapnic chronic respiratory insufficiency. Moreover, because of a growth restriction with oral feeding disorders, she underwent a percutaneous gastrostomy simultaneously with the third sclerotherapy procedure. During this same procedure a thulium laser treatment and an aryepiglottic fold resection were also performed. She is now 14 mo and she presents a motor delay with a mild axial hypotonia and still feeding disorder with a poor sucking reflex, that were considered as related to the long hospital stay.

Follow-up of sirolimus pharmacokinetics was initially performed twice a week to adjust the treatment with a targeted therapeutic concentration between 12 and 20 ng per ml. Difficulties were met to maintain the blood concentration in the therapeutic target. High concentrations (up to 43 ng per mL) were observed during the first 3 weeks of treatment, leading to successive adjustments of dosage. In the following months the serum concentration was measured weekly and was found to be between 5.7 and 19 ng per mL. The daily dosage of sirolimus was adapted following this concentration and fluctuated between 0.02 and 0.15 mg per kg. Safety of sirolimus administration was assessed by weekly monitoring of lipid tests and blood cell count. At 7 mo she developed mild dyslipidemia with a moderate increase of cholesterol and triglycerides levels which spontaneously normalized at 9 mo. So far, the blood cells counts are normal. Several infectious episodes were noticed but without serious presentation: she developed *Enterobacter cloacae* pneumonia during mechanical ventilation at 1 mo treated by intravenous antibiotics, a pyelonephritis at 5 mo without sign of sepsis nor bacteremia and an abdominal cellulitis around the gastrostomy tube at 8 mo without abscess and treated by oral antibiotics. She recovered as expected from these infections.

## Discussion

Through this case reporting we would like to illustrate and share our experience of an airway-threatening cystic lymphatic malformation in a preterm neonate, successfully managed using sirolimus as adjuvant therapy.

Cystic lymphatic malformations frequently occur in the cervicofacial area leading to a ventilatory support requirement. The emergency rests on the management of the acute respiratory distress syndrome at birth. This can be a challenge because of airway deviation and/or compression. That is why antenatal orientation of the pregnant women to a medical center with otorhinolaryngology and neonatal intensive care units is required for the safety of the early management of the baby. Moreover, glottis exposition and intubation can be facilitated by the use of video laryngoscopy, as reported in this case and a previous one (8). If possible, an EXIT procedure (endotracheal tube placement before cord section) can be considered in some cases. In our patient, the birth was planned at term in the referring hospital in the presence of the pediatric otorhinolaryngology specialist. However, this was not feasible because of the preterm labor: the baby was born in an hospital where there is no access to these procedures and was secondly transferred to the referring hospital.

Once ventilation is ensured, the second challenge is to define a strategy to manage this vascular mass. No guidelines are available and only a few cases are reported in the neonatal population. Treatments options are usually sclerotherapy, surgery, and medical treatment. In our case report, because of hemorrhagic cysts the sclerotherapy procedure was not as efficient as expected and because of the localization, surgery for debulking was not an option because it was considered as at high risk of tissue-damaging. Although an early administration of sirolimus in premature patient has barely been reported, the severity of the compression due to the mass led us to start such treatment in our patient at day 1 of life.

Mammalian target of rapamycin (mTOR) is a serin threonine kinase involved in endothelial and lymphatic cells proliferation and in cell survival, which goes through VEGF receptor-3 on the surface of lymphatic endothelium cells (9). Recently the use of sirolimus, an mTOR inhibitor, in cystic lymphatic malformations in newborns and infant has shown great results (10–13). In the present case the use of sirolimus in combination with sclerotherapy resulted in a reduction of mass size despite its slow action.

Sirolimus is usually associated with other treatment including sclerotherapy and/or surgery and the effects could be synergistic as it has been seen in a previous report (14). However, data are lacking to define the place of this drug in the management strategy.

Our observation also demonstrates that sirolimus can be well-tolerated, even in a preterm patient and even with such an early use. Side effects reported with a prolonged use of sirolimus include neutropenia, dyslipidemia, digestive disorders, lymphedema, abnormal wound healing, fatigue, etc. (15). In our patient, during the 14 months of administration, only a mild and transient cholesterol and triglycerides increase was noticed, spontaneously reversed. Moreover, a possible increase of infections in patients treated by sirolimus had previously been reported and some authors have considered antibiotic prophylaxis with trimethoprim-sulfamethoxazole in these patients. However, data are lacking about it since there is only a few cases concerning infants treated with sirolimus who developed pneumocystis and the major part of the literature data is about patients with solid transplant and/or receiving additional immunosupressive drugs (16). Here our patient developed three bacterial infections during her first year of life. These infections were not clinically severe and could not be clearly attributed to sirolimus exposure. Of note, these infections occurred during periods when the patient had serum level of sirolimus inside the therapeutic target. The choice of this therapeutic target was made in concentration with the local referring center for vascular malformations. However in the literature, the usual therapeutic target is frequently lower (2, 7, 10, 17). We can hypothesize that a lower serum level of sirolimus could have prevented from those adverse events in our patient.

Duration of treatment differed between all the studies found in the literature. Most of the patients had a long term treatment and were treated for several months. As the effects of sirolimus seems to appear after a few weeks/months of treatment, in most of the studies sirolimus was not withdrawn at last follow up (15).

## Conclusion

Sirolimus seems to be helpful in the management of cystic lymphatic malformations but without the necessary hindsight to define its place in the therapeutic strategy as well as its dosage and duration. Our observation along with a few other ones (6, 7, 10, 12) suggest that it could be considered as an adjuvant therapy in first line because it is a feasible therapy with an acceptable safety. This is particularly interesting in preterm neonates for whom surgery and sclerotherapy procedures can expose to severe complications. This therapy could also be considered when the location (larynx, base of the tongue) and/or the microcystic form are difficult to treat by surgery or sclerotherapy without side effects. Because of the small number of patients with cystic lymphatic malformations and because of the singularity of each case, it seems difficult to conduct randomized controlled trial to better define strategy of management of such malformation. Reporting relevant cases, both successful and unsuccessful, is therefore essential in documenting an optimal therapeutic approach in such a vulnerable population.

## Ethics Statement

Written informed consent was obtained from the minor(s)' legal guardian/next of kin for the publication of any potentially identifiable images or data included in this article.

## Author Contributions

MH, LV, LG, and KC: acquisition of the data. LG, SA, and MB: interpretation of the data. MH: draft of the article. LG, OC, and MB: revision of the manuscript. All authors: approved the final manuscript.

## Conflict of Interest

The authors declare that the research was conducted in the absence of any commercial or financial relationships that could be construed as a potential conflict of interest.
